# Supplementary material for: Identifying naturally occurring communities of primary care providers in the English National Health Service in London
Source: BMJ Open. 2020 Jul 20;10(7):e036504. doi: 10.1136/bmjopen-2019-036504 (PMC7375630; doi:10.1136/bmjopen-2019-036504)
Supplement: Author's manuscript [file bmjopen-2019-036504.draft_revisions.pdf]

BMJ Open

BMJ Open is committed to open peer review. As part of this commitment we make the peer review history of every article we publish publicly available.

When an article is published we post the peer reviewers' comments and the authors' responses online. We also post the versions of the paper that were used during peer review. These are the versions that the peer review comments apply to.

The versions of the paper that follow are the versions that were submitted during the peer review process. They are not the versions of record or the final published versions. They should not be cited or distributed as the published version of this manuscript.

BMJ Open is an open access journal and the full, final, typeset and author-corrected version of record of the manuscript is available on our site with no access controls, subscription charges or pay-per-view fees (http://bmjopen.bmj.com).

If you have any questions on BMJ Open's open peer review process please email info.bmjopen@bmj.com

BMJ Open

Identifying Naturally Occurring Communities of Primary Care Providers in the English National Health Service in London

Journal:	*BMJ Open*
Manuscript ID	bmjopen-2019-036504
Article Type:	Original research
Date Submitted by the Author:	18-Dec-2019
Complete List of Authors:	Clarke, Jonathan; Imperial College London, Centre for Health Policy; Imperial College London, Centre for Mathematics of Precision Healthcare<br>Beaney, Thomas; Imperial College of Science Technology and Medicine, Department of Primary Care<br>Majeed, Azeem; Imperial College, Primary Care<br>Darzi, Ara; Imperial College London,<br>Barahona, Mauricio; Imperial College London, Centre for Mathematics of Precision Healthcare; Imperial College London, Department of Mathematics
Keywords:	Health policy < HEALTH SERVICES ADMINISTRATION & MANAGEMENT, Organisation of health services < HEALTH SERVICES ADMINISTRATION & MANAGEMENT, PRIMARY CARE, PUBLIC HEALTH

SCHOLARONE™
Manuscripts

I, the Submitting Author has the right to grant and does grant on behalf of all authors of the Work (as defined in the below author licence), an exclusive licence and/or a non-exclusive licence for contributions from authors who are: i) UK Crown employees; ii) where BMJ has agreed a CC-BY licence shall apply, and/or iii) in accordance with the terms applicable for US Federal Government officers or employees acting as part of their official duties; on a worldwide, perpetual, irrevocable, royalty-free basis to BMJ Publishing Group Ltd ("BMJ") its licensees and where the relevant Journal is co-owned by BMJ to the co-owners of the Journal, to publish the Work in this journal and any other BMJ products and to exploit all rights, as set out in our licence.

The Submitting Author accepts and understands that any supply made under these terms is made by BMJ to the Submitting Author unless you are acting as an employee on behalf of your employer or a postgraduate student of an affiliated institution which is paying any applicable article publishing charge ("APC") for Open Access articles. Where the Submitting Author wishes to make the Work available on an Open Access basis (and intends to pay the relevant APC), the terms of reuse of such Open Access shall be governed by a Creative Commons licence – details of these licences and which Creative Commons licence will apply to this Work are set out in our licence referred to above.

Other than as permitted in any relevant BMJ Author's Self Archiving Policies, I confirm this Work has not been accepted for publication elsewhere, is not being considered for publication elsewhere and does not duplicate material already published. I confirm all authors consent to publication of this Work and authorise the granting of this licence.

For peer review only

Title:

Identifying Naturally Occurring Communities of Primary Care Providers in the English National Health Service in London

Authors:

Jonathan Clarke, Thomas Beaney, Azeem Majeed, Ara Darzi, Mauricio Barahona

Jonathan M Clarke
Clinical Research Fellow, Institute of Global Health Innovation, Imperial College London
Level 1, Faculty Building, South Kensington Campus, London, UK, SW7 2NA
ORCID ID: 0000-0003-1495-7746

Thomas E Beaney
Academic Clinical Fellow, Department of Primary Care and Public Health, Imperial College London
Level 2, Faculty Building, South Kensington Campus, London, SW7 2AZ, UK

Azeem Majeed
Professor of Primary Care, Department of Primary Care and Public Health, Imperial College London
Level 2, Faculty Building, South Kensington Campus, London, SW7 2AZ, UK

Ara W Darzi
Professor of Surgery, Institute of Global Health Innovation, Imperial College London
Level 1, Faculty Building, South Kensington Campus, London, SW7 2NA, UK

Mauricio Barahona
Professor of Mathematics, Department of Mathematics, Imperial College London
Huxley Building, South Kensington Campus, London, SW7 2AZ, UK

Correspondence to:
Jonathan Clarke
Clinical Research Fellow, Institute of Global Health Innovation, Imperial College London
Level 1, Faculty Building, South Kensington Campus, London, UK, SW7 2NA
j.clarke@imperial.ac.uk,
+44 (0) 7792 690037

Word Count: 3,416

For peer review only

Abstract

Objectives - Primary Care Networks (PCNs) are a new organisational hierarchy with wide-ranging responsibilities introduced in the NHS Long Term Plan. The vision is that they represent 'natural' communities of GPs with boundaries that make sense to practices, other healthcare providers and local communities. Our study aims to identify natural communities of GPs based on patient registration patterns using Markov Multiscale Community Detection, an unsupervised network-based clustering technique to create catchments for these communities.

Design - Retrospective observational study using Hospital Episode Statistics.

Setting – General practices in the 32 Clinical Commissioning Groups of Greater London

Participants - All adult patients resident in and registered to a GP practices in Greater London that had one or more outpatient encounters at NHS hospital trusts between 1st April 2017 and 31st March 2018.

Main outcome measures

The allocation of GP practices in Greater London to PCNs based on the registrations of patients resident in each Lower Super Output Area of Greater London. The population size and coverage of each proposed PCN.

Results - 3,428,322 unique patients attended 1,334 GPs in 4,835 LSOAs in Greater London. Our model grouped 1,291 GPs (96.8%) and 4,721 LSOAs (97.6%), into 165 mutually exclusive PCNs. The median PCN list size was 53,490, with a lower quartile of 38,079 patients and an upper quartile of 72,982 patients. A median of 70.1% of patients attended a GP within their allocated PCN, ranging from 44.6% to 91.4%.

Conclusions - With PCNs expected to take a role in population health management and with community providers expected to reconfigure around them, it is vital we recognise how PCNs represent their communities. Our method may be used by policy-makers to understand the populations and geography shared between networks.

For peer review only

Strengths and limitations of this study

- In the absence of data-driven approaches, Primary Care Networks have formed through interpersonal relationships between practices rather than through an understanding of the distribution of their registered patients.
- This study uses Markov Multiscale Community Detection, a data-driven, unsupervised clustering method to identify 'naturally occurring' communities of GP practices, which collectively form 165 Primary Care Networks across London.
- In doing so, this technique produces PCNs which are most representative of the spatial communities of patients for whom PCNs provide care.
- The proposed size of PCNs of 30,000 to 50,000 patients restricted to a single Clinical Commissioning Group may not represent patterns of care delivery in an urban setting.
- The use of Hospital Episode Statistics ensures that PCNs produced are related to secondary care utilisation, and therefore may not reflect those who rarely use healthcare services but remain registered to a GP practice.

For peer review only

Introduction

The introduction of Primary Care Networks in the NHS Long Term Plan in 2019 marks one of the biggest changes to general practice in England.[1] Since the NHS was formed, general practice has seen significant evolution from predominantly single-handed GPs, to large-scale businesses incorporating multi-disciplinary teams. This trend has resulted in a corresponding decline in the number of practices over time to 6,984 practices in England in March 2019 with an increase in the average list size to 8,550 patients.[2] In parallel, different organisational hierarchies have developed and coexist, linking together practices within a defined geography. Currently, many GP practices collaborate informally as Federations, while all GP practices are now part of larger Clinical Commissioning Groups (CCGs). More recently, Sustainability and Transformation Partnerships have formed through collaboration between CCGs. The new NHS Long Term Plan and GP contract, announced in 2019, introduced the 'Primary Care Network' (PCN),[1,3,4] a major new organisational structure operating at a population size smaller than the CCG. PCNs are designed to provide economies of scale, without losing the community focus of general practice. In contrast to the informal arrangement of GP Federations, PCNs represents a policy shift towards more formal collaboration between GP practices at a smaller scale than CCGs. This new organisational structure comes with significant financial incentives—an additional £1.8 billion in the form of Directed Enhanced Services has been earmarked to recruit additional staff and expand the role of primary care.[5] Two of the key roles of PCNs will be to integrate community care providers within the network, and to take on an increasing role in the population health management of their communities. These objectives highlight the importance of properly delineating geographically contiguous areas to ensure the efficiency of services provided by PCNs.[3]

While many practices are already members of networks or federations, or informally collaborate with other GPs, the new organisational structure may not match existing arrangements. In terms of their size, PCNs are specified as networks of neighbouring practices covering a population of 30,000 to 50,000, forming smaller clusters than CCGs. In terms of their borders, the contract specification states that, typically, PCNs will not cross the boundaries of a CCG, although this is not an absolute requirement.[3] The vision from NHS England is that PCNs serve 'natural communities' and that their boundaries make sense to their practices, community-based providers, and to their local communities.[6] In reality, boundaries that make sense to all three groups may be difficult to harmonise, particularly where patients are free to choose a GP outside their geographical area, but what might these natural communities look like in the absence of external influence?

In this article, we set out an approach to defining communities that conform to the criteria required of PCNs, based only on the registration of patients from a given geographical area to a GP. Our approach uses Markov Multiscale Community Detection (MMCD), which uses Louvain optimisation to detect and obtain robust partitions of a network, without imposing the number of partitions that should be

For peer review only - http://bmjopen.bmj.com/site/about/guidelines.xhtml

produced. Briefly, MMCD is an unsupervised graph-based clustering method that exploits how a diffusion process spreads on a network to identify communities of nodes that share information consistently over different time scales. In this case, the nodes are GP practices and the communities correspond to practices that have stronger connections with one another than to other nodes in the network. These techniques have previously been applied to the organisation of planned orthopaedic care in England and, in other disciplines, to identify communities within processes as diverse as metabolic networks, transport systems and power grids.[7–11] This study aims to use these unsupervised network analysis techniques to identify underlying communities of GP practices who share patients from the same LSOAs of residence as one another. The ensuing 'GP communities' are thus identified in an unsupervised manner based on patient registration data. In doing so, it seeks to produce and then understand data-driven PCNs within London that meet the ambitions of policy makers to create 'natural' communities of primary care that represent their local populations.

Methods

All adult patients presenting to outpatient secondary care in England from 1st April 2017 to 31st March 2018 were identified from Hospital Episode Statistics (HES).[12] The Lower Super Output Area (LSOA) of residence of the patient and the unique identifier of their registered GP practice were identified from these records. In cases where a patient was registered at more than one LSOA and GP practice combination within the overall time period, the record with the highest frequency for that individual was chosen; where these were tied, the most recent combination was chosen. GP practices contributing fewer than 100 patients were excluded.

The equivalent market size (EMS) of each LSOA was calculated as the reciprocal of the Herfindahl Hirschman Index (Equation 1):

$$HHI = \sum_{i=1}^{N} s_i^2 \qquad (1)$$

Equation 1: Herfindahl-Hirschman Index of an LSOA. Here s_i is the proportion of patients from an LSOA registered to GP practice i, and N is the number of GP practices in the dataset.

The EMS represents the number of GP practices to which members of an LSOA would be registered if each practice occupied an equal market share of registrations. It therefore provides an estimate of the concentration of the market for primary care registrations within each LSOA.[13–15] The probability of a patient from each LSOA being registered to a GP practice was calculated, and a normalised cosine similarity matrix of GP practices was computed (Equation 2).

For peer review only

$$similarity_{AB} = \frac{\sum_{i=1}^{n} A_i B_i}{\sqrt{\sum_{i=1}^{n} A_i^2}\sqrt{\sum_{i=1}^{n} B_i^2}} \qquad (2)$$

Equation 2: Calculation of cosine similarity between GP practices. Here A_i is the proportion of patients from GP practice A resident in LSOA i, B_i is the proportion of presentations from GP practice B resident in LSOA I, and n is the total number of LSOAs in the dataset.

The similarity matrix can be thought of as the adjacency matrix of a dense, undirected, weighted network connecting GPs to one another according to the similarity of their patterns of patient registration across LSOAs. This network was sparsened using the Relaxed Minimum Spanning Tree (RMST) technique, a method used elsewhere in applied network science to sparsen a dense, inhomogeneous network to preserve both local and global connectivity within a network.[16–18] The sparsened network was subsequently partitioned using Markov Multiscale Community Detection (MMCD) to produce partitions of the GPs according to shared patterns of registration from LSOAs. Each of these partitions corresponds to a 'natural' community directly derived from the registration data.[7,19–21]

MMCD was performed over a range of 400 Markov times from 0.1 to 1 and over a range of 40 RMST pruning parameters from 2.1 – 6.0. Each of these 16,000 models, was repeated 500 times and the most common network partition from each model was selected.

For each of the 16,000 partitions produced, the pairwise distances between all practices within each PCN was calculated. Where the median distance from a practice to all other practices within a PCN community was more than four times the median pairwise distance between all practices within the PCN community, this practice was excluded as a spatial outlier. The number of practices within each PCN community was calculated and the proportion of practices contained within the polygon drawn around the outer geographic limits of the practices comprising the PCN community was calculated.

PCN communities where the number of practices was less than three or greater than twenty, or where the percentage of practices spatially located within the outer spatial limits of the PCN community was more than 25% were excluded. The total number of practices present in the remaining PCN communities was calculated. The partition with the highest number of included GP practices was taken as the optimal partition.

For this optimal partition, the GP practice name, location and CCG were linked by practice code, using data from NHS Digital. Practice list sizes as of March 2018 were also linked using data from NHS

For peer review only

Digital.[22] Where a practice list size was not available, such as in cases where a practice had closed or merged over the time period, list sizes as of April 2017 were used.[23]

LSOAs were subsequently assigned to a PCN community based on the PCN to which the highest number of patients within a given LSOA were registered. GP practices were mapped along with their corresponding assigned community allocation and LSOA boundaries. The proportion of patients resident within the same PCN as their registered GP was calculated for each PCN.

Patient and Public Involvement (PPI) Statement

The data on which this study is based was granted following review by a panel including patient and lay representatives. Patients were not invited to contribute to the writing or editing of this document for readability or accuracy.

Results

A total of 3,428,322 unique adult patients resident in London attended a total of 20,173,937 outpatient appointments to NHS hospitals between 1st April 2017 and 31st March 2018 (Figure 1). All 4,835 LSOAs in London were represented. A total of 1,334 GP practices were identified within London belonging to a total of 32 Clinical Commissioning Groups.

Residents in the same LSOA in London were generally registered to a wide range of GP practices. Figure 2 demonstrates the equivalent market size of LSOAs across London with respect to GP practices. A wide range of equivalent market size was seen, from 1.1 to 20.6. 185 LSOAs (3.8%) had an equivalent market size of less than two GP practices, consistent with primary care provision by a single dominant GP practice. The median equivalent market size across London was 4.9 GP practices per LSOA, while 259 LSOAs (5.4%) had an equivalent market size of more than ten GP practices.

The CCGs with the highest median LSOA-level equivalent market size were found in the peripheral CCGs of North London in Hounslow (7.0), Barking and Dagenham (6.7) and Brent (6.5). The lowest median LSOA-level equivalent market sizes were identified in Tower Hamlets (3.2), Lewisham (3.7) and Merton (3.8). Overall, the median equivalent market size for LSOAs north of the river Thames was 23% higher than those south of the river (5.3 vs. 4.3). The dilution of primary care markets also differed greatly between adjacent CCGs. Crossing the CCG boundary from Tower Hamlets to Newham resulted in a 100% increase in equivalent market size, while crossing from Richmond to Hounslow resulted in an 80% increase. LSOAs with a higher proportion of patients over 65 years of age had significantly smaller market sizes, with a 10% increase in the absolute proportion of over 65s resulting in a reduction in market size of 0.5 practices in a univariate linear regression model ($P < 0.001$).

For peer review only

An optimal configuration of GP communities was found at an RMST parameter of 5.5 and timestep of 148. For this clustering, only 43 GP practices (out of a total of 1,334) were unassigned to a community according to our criteria: 28 practices were unassigned as they lay in 20 communities with only 1-2 practices, whereas a further 15 practices were unassigned as they were spatial outliers. Collectively, these 43 practices were the modal provider of primary care for 114 LSOAs with a total population of 187,101 (2.3%). This optimal partition consisted of 165 PCNs grouping 1,291 practices, covering 4,721 LSOAs in London and 97.7% of the estimated London population. A map of this optimal configuration, displaying GP practices superimposed on the LSOAs assigned to each PCN is shown in Figure 3. The excluded communities, represented by dashed lines, are predominantly situated peripherally or bordering the river Thames.

The PCNs ranged in size from 3 to 18 practices per community with median list size of 53,490 patients (ranging from 14,574 to 176,982 patients, with a lower quartile of 38,079 and upper quartile of 72,982 patients). Around two thirds (67.9%) of PCNs contained practices from only one CCG and the remaining PCNs contained practices from either two (23.0%) or three (9.1%) CCGs.

Across all 4,721 LSOAs, the median percentage of patients registered to GP practices located within their allocated PCN was 73.7%, ranging from a minimum of 24.8% to a maximum 98.6% (Figure 4, left). Across the 165 PCNs, a median of 70.1% of patients within the PCN catchment area were registered to a GP practice within the PCN, ranging from a minimum of 44.6% to a maximum of 91.4% (Figure 4, right). The median Herfindahl-Hirschman Index of the LSOAs was 0.57, and there was a median equivalent market size of 1.75 PCNs for each LSOA.

Discussion

As the health system develops to prioritise integrated and collaborative working, we need to improve our understanding of the relationships between providers and patients. With the requirement for GPs to form larger organisational units in the form of PCNs, there is a need to quantify how representative these communities are of their populations. The techniques used in our study provide an unsupervised, data-driven means of producing mutually exclusive PCNs formed by bringing together GP practices that frequently provide care to patients from the same geographic regions. In doing so, it makes no assumptions regarding geography or organisational hierarchies and produces 'natural' communities. Using this technique, we showed that despite having no prior geographic knowledge, 97.7% of the population of London may be assigned to a PCN of appropriate size and which are spatially consistent.

The composition of PCNs

The factors that determine practices joining a PCN are complex and rely on the interpersonal and professional relationships between GPs as much as the shared relationships in registration of the local

For peer review only

population. Furthermore, pre-defined boundaries cannot be ignored, with local authorities and CCGs taking responsibility for commissioning for a given geographical area and registered patients. However, networks must work for their population, and existing tools to guide decision-making are limited. While most practices have now joined PCNs, it is likely that boundaries will change as practices move in and out of PCNs, reflecting the complex dynamics of a system which needs to work for the population and providers.

In the context of the PCNs that are forming across London, our findings may in the future be directly compared with the actual PCNs that form. While it is hoped that the PCNs suggested by this study conform to those being formed across London, identifying discrepancies between the two conformations may offer a means of identifying PCNs which may not adequately represent local patterns of patient registration, and therefore may benefit from reorganisation. Secondly, where practices struggle to align with one another to form PCNs, the findings of this study offer a tool by which to propose suitable PCN structures.

Our findings also raise questions regarding the optimal size and configuration of PCNs across London. Our modelling suggests that the 30,000 to 50,000 recommended list size for PCNs may be too restrictive. The finding of a median list size for our optimal configuration of 53,490 in London, with an interquartile range from 38,000 to 73,000 patients suggests the current recommended range may not permit larger networks to form where underlying patient registration patterns would favour this. Similarly, while a clear majority of the PCNs we found (67.9%) are formed from practices within a single CCG, almost one third contain practices in two or three CCGs , suggesting that there should be flexibility in conformity to CCG boundaries. However, there may be additional problems for a PCN containing practices from more than one CCG where priorities do not align.

The role of PCNs

If PCNs take on a role in population health management, and community services are required to reconfigure to match the footprints of PCNs,[24,25] then the need to understand the demographics and geography of PCNs becomes critical. Through our network analysis method, we defined an approach to assign catchment areas and estimate the catchment population for each PCN. We found that on average, almost one third of the population living within the PCN catchment area attended a practice outside of the PCN, which was as high as half of the population for some PCNs. Understanding the dynamics of registration patterns in the local population could thus have significant implications on the design of place-based services.

Population dispersion is likely to be a greater problem in a densely populated urban area such as London, where there are extensive transport links and a greater choice of providers within a shorter commuting

For peer review only

distance. However, our estimates, which by design are modelled to represent optimal patterns of registration, are likely to overestimate coverage of the PCN compared to those PCNs that have already formed. The opportunity for patients to retain registration with a practice after moving outside of its historic catchment postcodes may reduce coverage of a PCN where a fixed relationship to a discrete geographic area is required.

The emergence of digital NHS general practice, in the form of GP at Hand and its peers, disrupts the notion of a 'place-based' relationship between primary care providers and geographic communities of patients. Currently, the registered population for such services is disproportionately of working age, with 98.5% of registrants between 20-64 years of age, compared to a London-wide estimate of 75.3%.[26,27] The proportion of patients with one or more long term conditions using this service is almost half that of London-wide estimates (10.6% compared to 18.3%).[26,28] Therefore, patients registered with digital general practices are less likely to require the place-based services and community integration which underpins the establishment of Primary Care Networks.

As such innovations in registration and GP provision mature and potentially scale across the health system, the significant differences between patients using digital primary care services and traditional general practices may undermine efforts to enforce relationships between primary care providers and discrete geographic communities of patients. This trend may signal the emergence of a differentiated system of primary care, where those with low care needs are served by essentially rootless digital primary care providers and those with higher care needs are attended to by well-integrated, accessible primary care providers with a nearby physical presence. In such cases, the creation of Primary Care Networks by providers of digital primary care may be orthogonal to the underlying ethos of vertical integration and investment in relationships with community care providers that underlies the current policy.

Limitations

One of the main limitations of our analysis is in use of secondary care, rather than primary care data. While HES represented 3.4 million patients in London, this covers less than half of the estimated population of 8.8 million.[25,29] Patients in HES have, by definition, used secondary care services at least once over the study period, and may not be representative of the whole population registered with primary care. Older people, and those with a higher number of chronic conditions are more likely to be represented in HES, and these patients may have different criteria in choosing a GP, perhaps favouring closer proximity. This is supported by the finding in this study that LSOAs with higher proportions of people over the age of 65 have significantly lower equivalent market sizes. Use of GP registration data would be preferable, but is not readily available and was outside the scope of the current study.

For peer review only

A limitation with our method was that the optimal configuration was unable to fit every practice in London to a PCN, with 43 (out of 1,334) unassigned as a result of our selection criteria. 15 practices were unassigned as spatial outliers, which is to say that their median distance to all other practices in their network was more than four times greater than the median pairwise distance between all practices in the network. These rare instances may reflect the statistical noise of the modelling technique which is agnostic to the spatial proximity of providers to one another. A further 28 practices were unassigned due to their proposed PCNs containing fewer than three practices. In these cases, allocation of unassigned practices to nearby larger PCNs could be an appropriate solution to ensure complete allocation of practices to PCNs. The finding that many unassigned practices were near the periphery of London suggests a boundary effect where the exclusion of practices and the population outside of London may have affected the model in these regions.

Conclusion

As health systems adapt towards closer integration across services, network analysis offers a data-driven and unbiased means of understanding the connections between Primary Care Networks and their patients. With the growth of online GP consultation providers, such as that provided by GP at Hand in London, the traditional model of general practice as a place-based provider rooted in the local community is being challenged. Upscaling primary care into larger networks has the potential to weaken further the ties between providers and their communities. There is a pressing need to better understand how these networks will represent their geographies and patients, to identify who may gain and who may lose out, and ensure a well-intentioned policy does not widen inequalities in health.

For peer review only

Author Contributions

JC and TB were involved in all aspects of the study. MB was involved in the development of the methodology and assisted in the formal analysis. MB, AM and AD were involved in the conceptualization of the study and in the reviewing and editing of the draft. JC has had access to all the data in the study and all authors had final responsibility for the decision to submit for publication. JC attests that all listed authors meet authorship criteria and that no others meeting the criteria have been omitted.

Acknowledgements and Competing Interests

All authors have completed the ICMJE uniform disclosure form at www.icmje.org/coi_disclosure.pdf and declare: JC, TB, AD and MB have no competing interests to declare. AM is a GP Principal in an NHS general practice.

This article is independent research supported by grants from The Peter Sowerby Foundation and the National Institute for Health Research (NIHR) Imperial Patient Safety and Translational Research Centre (PSTRC) PSTRC_2016_004. Infrastructure support for this work was provided by the NIHR Imperial Biomedical Research Centre (BRC) 1215-20013. TB is supported by a National Institute for Health Research (NIHR) Academic Clinical Fellowship. TB acknowledges support from the NIHR Imperial Biomedical Research Centre (BRC) and the NIHR Applied Research Collaboration for NW London. AM is supported by the NIHR Applied Research Collaboration for NW London. MB acknowledges support from EPSRC grant EP/N014529/1 supporting the EPSRC Centre for Mathematics of Precision Healthcare. Data management was provided by the Big Data and Analytical Unit (BDAU) at the Institute of Global Health Innovation (IGHI), Imperial College London. The views expressed in this publication are those of the author(s) and not necessarily those of the NHS, the National Institute for Health Research or the Department of Health.

For peer review only

Data Sharing Statement

Data used in this study were obtained from NHS Digital for the purpose of this work and may only be accessed through direct application to NHS Digital. Patient-level data is required for the analyses conducted, and therefore sharing of data pertaining to this study is not possible. Data for primary care network assignments are available from the authors on request.

Dissemination declaration

Data as to GP practice-level allocation to putative Primary Care Networks will be made publicly available on www.healthdatascience.co.uk and have been disseminated to local primary care providers and commissioners.

Transparency declaration

The lead author affirms that this manuscript is an honest, accurate, and transparent account of the study being reported; that no important aspects of the study have been omitted; and that any discrepancies from the study as planned have been explained.

Statement of Ethical Approval

This study received local ethical approval through the Imperial College Research Ethics Committee (17IC4178).

For peer review only

Figures:

Figure 1: Algorithm for participant selection from Hospital Episode Statistics

Figure 2: Equivalent market size of general practices for all LSOAs in London.

Figure 3: Optimal configuration of general practice communities in London obtained from our model, with LSOAs assigned to most common provider. Dashed lines represent unassigned communities.

Figure 4: Left: proportion of patients within each LSOA attending the modal PCN. Right: proportion of patients within the LSOAs assigned to each PCN attending a practice within the PCN. Dashed lines represented unassigned communities

For peer review only

References:

1. NHS England. *The NHS Long Term Plan*. (2019).
2. NHS Digital. Patients registered at a GP Practice - March 2019. *Online, NHS Digital* (2019).
3. NHS England & British Medical Association. *Investment and evolution : A five-year framework for GP contract reform to implement The NHS Long Term Plan*. (2019).
4. Baird, B. Primary care networks explained. *The King's Fund* (2019).
5. NHS England. *Network Contract Directed Enhanced Service*. (2019).
6. NHS England. Primary care networks. (2019).
7. Schaub, M. T., Delvenne, J. C., Yaliraki, S. N. & Barahona, M. Markov dynamics as a zooming lens for multiscale community detection: Non clique-like communities and the field-of-view limit. *PLoS One* **7**, 1–20 (2012).
8. Schaub, M. T., Lehmann, J., Yaliraki, S. N. & Barahona, M. Structure of complex networks: Quantifying edge-to-edge relations by failure-induced flow redistribution. *Netw. Sci.* **2**, 1–24 (2014).
9. Beguerisse-Díaz, M., Bosque, G., Oyarzún, D., Picó, J. & Barahona, M. Flux-dependent graphs for metabolic networks. 1–25 (2016).
10. Clarke, J. M., Barahona, M. & Darzi, A. W. Defining Hospital Catchment Areas Using Multiscale Community Detection: A Case Study for Planned Orthopaedic Care in England. *bioRxiv* 619692 (2019). doi:10.1101/619692
11. Schaub, M. T., Lambiotte, R. & Barahona, M. Encoding dynamics for multiscale community detection: Markov time sweeping for the map equation. *Phys. Rev. E - Stat. Nonlinear, Soft Matter Phys.* **86**, 1–10 (2012).
12. NHS Digital. Hospital Episode Statistics (HES).
13. Clarke, J. M., Warren, L. R., Arora, S., Barahona, M. & Darzi, A. W. Guiding interoperable electronic health records through patient-sharing networks. *npj Digit. Med.* **1**, 65 (2018).
14. Rhoades, S. A. Market share inequality, the HHI, and other measures of the firm-composition of a market. *Rev. Ind. Organ.* **10**, 657–674 (1995).
15. Feng, Y. *et al.* Association between market concentration of hospitals and patient health gain following hip replacement surgery. *J. Health Serv. Res. Policy* **20**, 11–7 (2015).
16. Amor, B. *et al.* Community detection and role identification in directed networks: understanding the Twitter network of the care.data debate. *arXiv.org* **1508**, 3165 (2015).
17. Altuncu, M. T., Mayer, E., Yaliraki, S. N. & Barahona, M. From free text to clusters of content in health records: an unsupervised graph partitioning approach. *Appl. Netw. Sci.* **4**, 2 (2019).
18. Beguerisse-Diaz, M., Vangelov, B. & Barahona, M. Finding role communities in directed networks using Role-Based Similarity, Markov Stability and the Relaxed Minimum Spanning Tree. *2013 IEEE Glob. Conf. Signal Inf. Process. Glob. 2013 - Proc.* 937–940 (2013).

For peer review only

doi:10.1109/GlobalSIP.2013.6737046

19. Delvenne, J.-C., Schaub, M. T., Yaliraki, S. N. & Barahona, M. The Stability of a Graph Partition: A Dynamics-Based Framework for Community Detection. in *Dynamics On and Of Complex Networks, Volume 2*. 221–242 (Birkhäuser, New York, NY, 2013). doi:10.1007/978-1-4614-6729-8_11
20. Delvenne, J.-C., Yaliraki, S. N. & Barahona, M. Stability of graph communities across time scales. *Proc. Natl. Acad. Sci. U. S. A.* **107**, 12755–60 (2010).
21. Lambiotte, R., Delvenne, J.-C. & Barahona, M. Laplacian Dynamics and Multiscale Modular Structure in Networks. 1–29 (2008). doi:10.1109/TNSE.2015.2391998
22. NHS Digital. Patients Registered at a GP Practice, March 2018. *Online, NHS Digital* (2018).
23. NHS Digital. Numbers of Patients Registered at a GP Practice - April 2017. *Online, NHS Digital* (2017).
24. Murray, R. Primary care networks and community health services: further, faster! *The King's Fund* (2019).
25. Murray, R. Primary care networks and the NHS long-term plan: the new player on the pitch. *The King's Fund* (2019).
26. Commission, C. Q. *GP at hand: Inspection report*.
27. Statistics, O. for N. Mid-year Population Estimates 2019 - Office for National Statistics. Available at: https://www.ons.gov.uk/peoplepopulationandcommunity/populationandmigration/populationestimates. (Accessed: 26th October 2019)
28. England, N. *London - A Call to Action*.
29. Greater London Authority. GLA population and Household Projections - Central Trend (population, 2017-based). (2017).

For peer review only

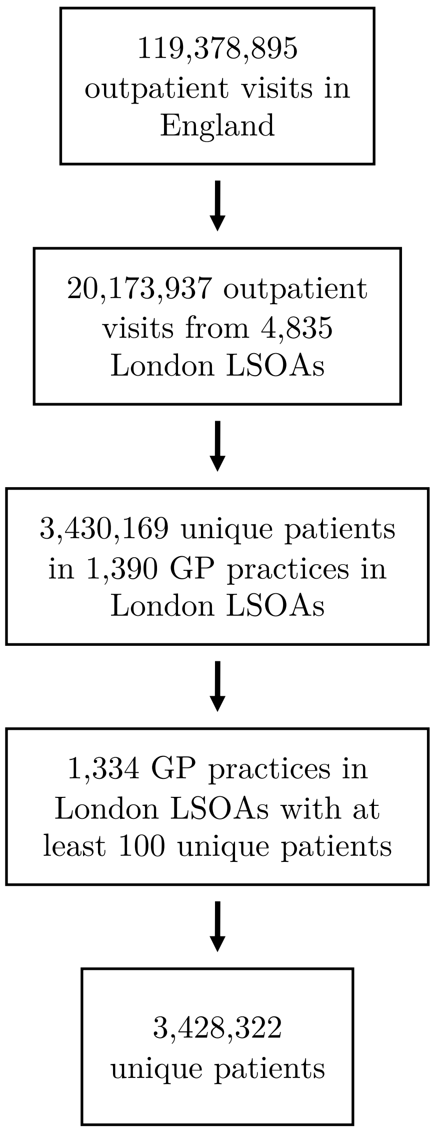
119,378,895 outpatient visits in England
20,173,937 outpatient visits from 4,835 London LSOAs
3,430,169 unique patients in 1,390 GP practices in London LSOAs
1,334 GP practices in London LSOAs with at least 100 unique patients
3,428,322 unique patients

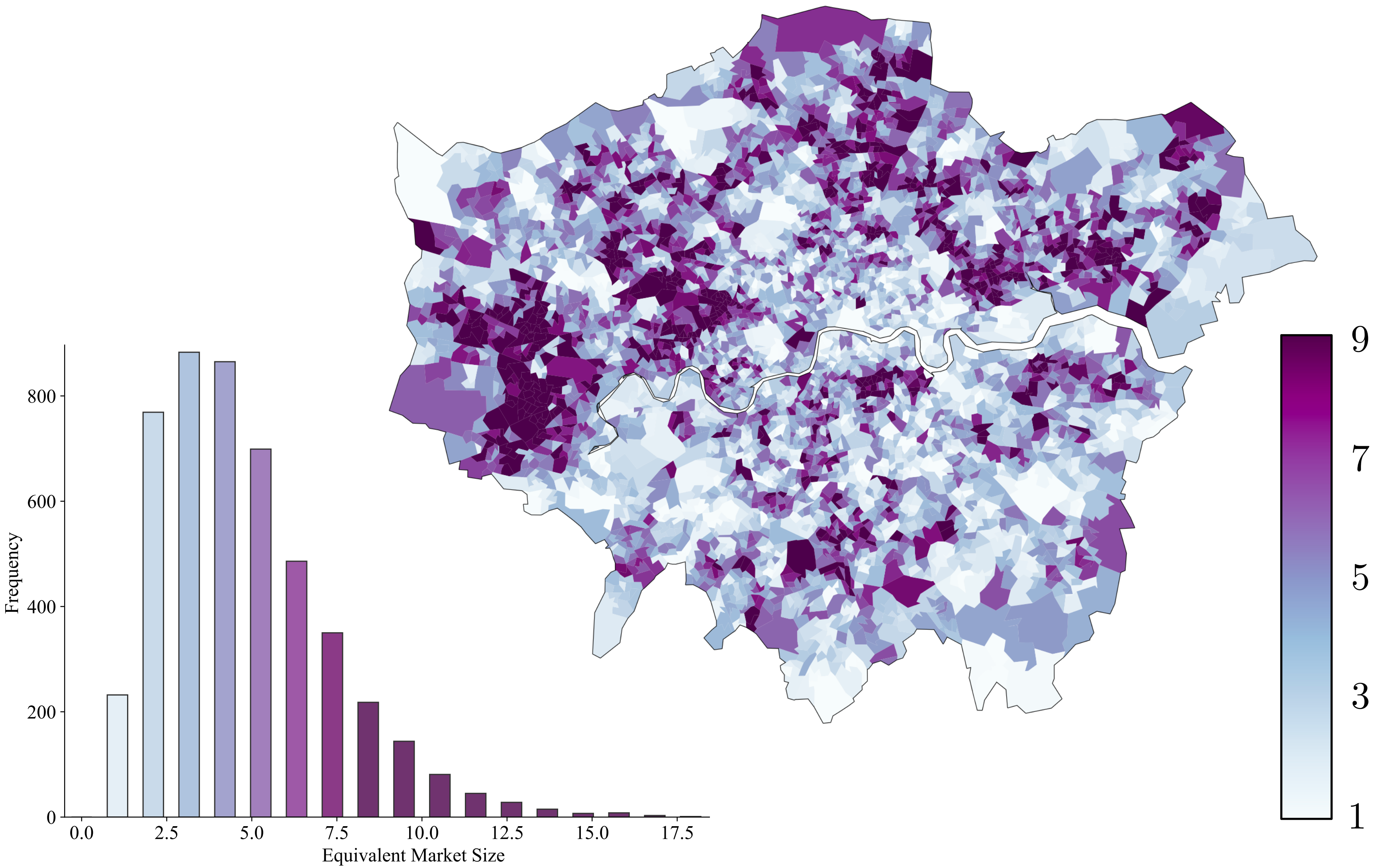
800
600
400
200
0
Frequency
0.0
2.5
5.0
7.5
10.0
12.5
15.0
17.5
Equivalent Market Size
9
7
5
3
1

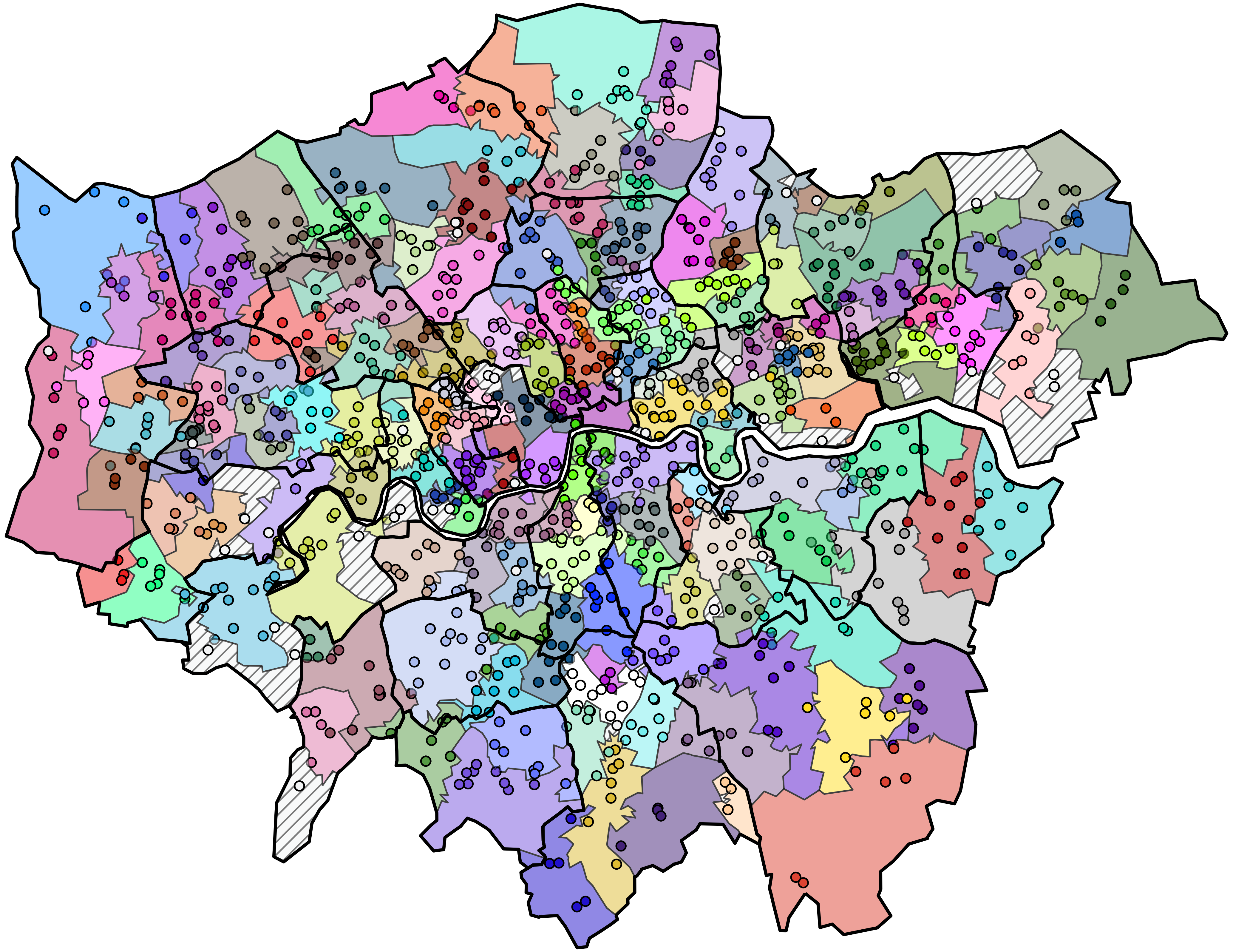

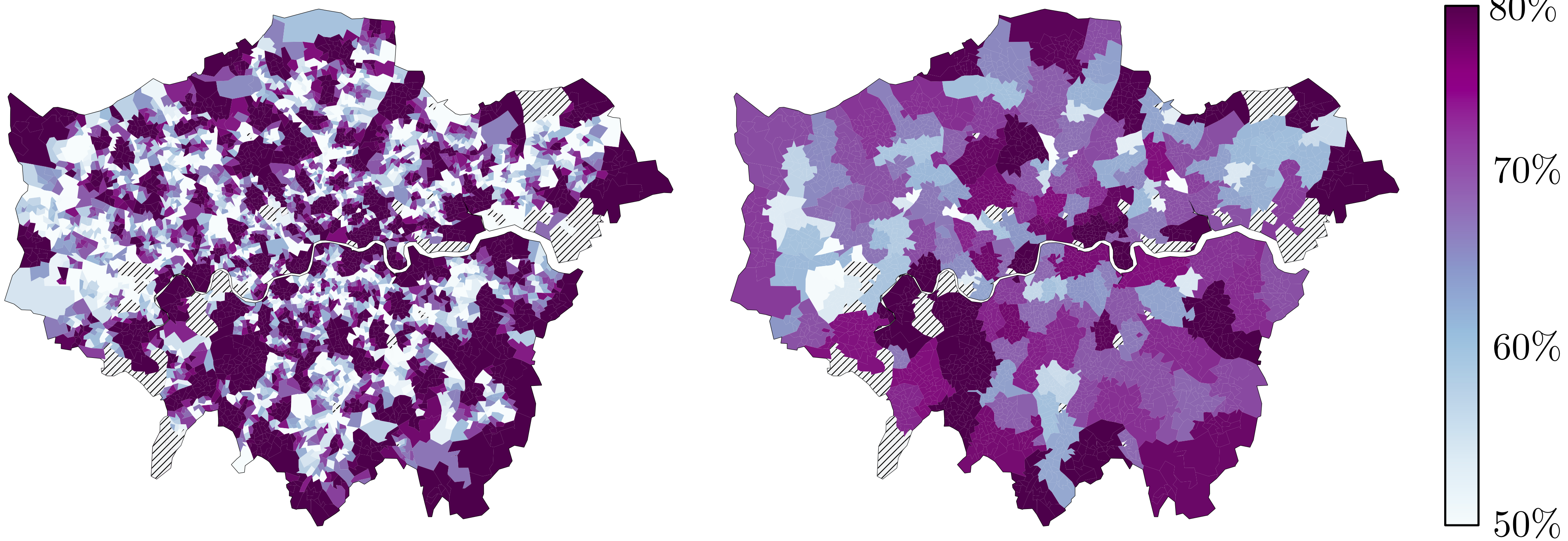
80%
70%
60%
50%

The RECORD statement – checklist of items, extended from the STROBE statement, that should be reported in observational studies using routinely collected health data.

	Item No.	STROBE items	Location in manuscript where items are reported	RECORD items	Location in manuscript where items are reported
**Title and abstract**					
	1	(a) Indicate the study's design with a commonly used term in the title or the abstract (b) Provide in the abstract an informative and balanced summary of what was done and what was found	Abstract	RECORD 1.1: The type of data used should be specified in the title or abstract. When possible, the name of the databases used should be included.<br><br>RECORD 1.2: If applicable, the geographic region and timeframe within which the study took place should be reported in the title or abstract.<br><br>RECORD 1.3: If linkage between databases was conducted for the study, this should be clearly stated in the title or abstract.	Abstract (methods)<br><br>Abstract (setting / participants)<br><br>N/A
**Introduction**					
Background rationale	2	Explain the scientific background and rationale for the investigation being reported	Introduction		
Objectives	3	State specific objectives, including any prespecified hypotheses	Introduction		
**Methods**					
Study Design	4	Present key elements of study design early in the paper	Methods		
Setting	5	Describe the setting, locations, and relevant dates, including periods of recruitment, exposure, follow-up, and data collection	Methods		

Participants	6	*(a) Cohort study* - Give the eligibility criteria, and the sources and methods of selection of participants. Describe methods of follow-up<br>*Case-control study* - Give the eligibility criteria, and the sources and methods of case ascertainment and control selection. Give the rationale for the choice of cases and controls<br>*Cross-sectional study* - Give the eligibility criteria, and the sources and methods of selection of participants<br><br>*(b) Cohort study* - For matched studies, give matching criteria and number of exposed and unexposed<br>*Case-control study* - For matched studies, give matching criteria and the number of controls per case	N/A<br><br>N/A<br><br>Methods<br><br>N/A<br><br>N/A	RECORD 6.1: The methods of study population selection (such as codes or algorithms used to identify subjects) should be listed in detail. If this is not possible, an explanation should be provided.<br><br>RECORD 6.2: Any validation studies of the codes or algorithms used to select the population should be referenced. If validation was conducted for this study and not published elsewhere, detailed methods and results should be provided.<br><br>RECORD 6.3: If the study involved linkage of databases, consider use of a flow diagram or other graphical display to demonstrate the data linkage process, including the number of individuals with linked data at each stage.	Methods<br><br>Validation of Markov Multiscale Community Detection cited<br><br>N/A
Variables	7	Clearly define all outcomes, exposures, predictors, potential confounders, and effect modifiers. Give diagnostic criteria, if applicable.	Methods	RECORD 7.1: A complete list of codes and algorithms used to classify exposures, outcomes, confounders, and effect modifiers should be provided. If these cannot be reported, an explanation should be provided.	Methods
Data sources/ measurement	8	For each variable of interest, give sources of data and details of methods of assessment (measurement).<br>Describe comparability of assessment methods if there is more than one group	Methods		

For peer review only

Bias	9	Describe any efforts to address potential sources of bias	Discussion		
Study size	10	Explain how the study size was arrived at	Methods		
Quantitative variables	11	Explain how quantitative variables were handled in the analyses. If applicable, describe which groupings were chosen, and why	Methods		
Statistical methods	12	(a) Describe all statistical methods, including those used to control for confounding<br>(b) Describe any methods used to examine subgroups and interactions<br>(c) Explain how missing data were addressed<br>(d) *Cohort study* - If applicable, explain how loss to follow-up was addressed<br>*Case-control study* - If applicable, explain how matching of cases and controls was addressed<br>*Cross-sectional study* - If applicable, describe analytical methods taking account of sampling strategy<br>(e) Describe any sensitivity analyses	N/A<br>Methods<br>Discussion<br>N/A<br>N/A<br>Methods, Discussion<br>N/A		
Data access and cleaning methods		..		RECORD 12.1: Authors should describe the extent to which the investigators had access to the database population used to create the study population.	Declaration of interests

For peer review only

				RECORD 12.2: Authors should provide information on the data cleaning methods used in the study.	Methods
Linkage		..		RECORD 12.3: State whether the study included person-level, institutional-level, or other data linkage across two or more databases. The methods of linkage and methods of linkage quality evaluation should be provided.	Methods
**Results**					
Participants	13	(a) Report the numbers of individuals at each stage of the study (*e.g.*, numbers potentially eligible, examined for eligibility, confirmed eligible, included in the study, completing follow-up, and analysed)<br>(b) Give reasons for non-participation at each stage.<br>(c) Consider use of a flow diagram	Results<br><br>Discussion<br><br>Figure 1	RECORD 13.1: Describe in detail the selection of the persons included in the study (*i.e.,* study population selection) including filtering based on data quality, data availability and linkage. The selection of included persons can be described in the text and/or by means of the study flow diagram.	Methods, Figure 1
Descriptive data	14	(a) Give characteristics of study participants (*e.g.*, demographic, clinical, social) and information on exposures and potential confounders<br>(b) Indicate the number of participants with missing data for each variable of interest<br>(c) *Cohort study* - summarise follow-up time (*e.g.*, average and total amount)	Results<br><br>Results<br><br>N/A		
Outcome data	15	*Cohort study* - Report numbers of outcome events or summary measures over time<br>*Case-control study* - Report numbers in each exposure	N/A<br><br>N/A		

For peer review only

		category, or summary measures of exposure<br>*Cross-sectional study* - Report numbers of outcome events or summary measures	Results		
Main results	16	(a) Give unadjusted estimates and, if applicable, confounder-adjusted estimates and their precision (e.g., 95% confidence interval). Make clear which confounders were adjusted for and why they were included<br>(b) Report category boundaries when continuous variables were categorized<br>(c) If relevant, consider translating estimates of relative risk into absolute risk for a meaningful time period	Results<br>N/A<br>N/A		
Other analyses	17	Report other analyses done—e.g., analyses of subgroups and interactions, and sensitivity analyses	Results		
**Discussion**					
Key results	18	Summarise key results with reference to study objectives	Discussion		
Limitations	19	Discuss limitations of the study, taking into account sources of potential bias or imprecision. Discuss both direction and magnitude of any potential bias	Discussion	RECORD 19.1: Discuss the implications of using data that were not created or collected to answer the specific research question(s). Include discussion of misclassification bias, unmeasured confounding, missing data, and changing eligibility over time, as they pertain to the study being reported.	Discussion
Interpretation	20	Give a cautious overall interpretation of results considering objectives,	Discussion		

For peer review only

		limitations, multiplicity of analyses, results from similar studies, and other relevant evidence			
Generalisability	21	Discuss the generalisability (external validity) of the study results	Discussion		
**Other Information**					
Funding	22	Give the source of funding and the role of the funders for the present study and, if applicable, for the original study on which the present article is based	Included after conclusions of manuscript		
Accessibility of protocol, raw data, and programming code		..		RECORD 22.1: Authors should provide information on how to access any supplemental information such as the study protocol, raw data, or programming code.	Included after conclusions of manuscript

*Reference: Benchimol EI, Smeeth L, Guttmann A, Harron K, Moher D, Petersen I, Sørensen HT, von Elm E, Langan SM, the RECORD Working Committee. The REporting of studies Conducted using Observational Routinely-collected health Data (RECORD) Statement. *PLoS Medicine* 2015; in press.

*Checklist is protected under Creative Commons Attribution (CC BY) license.

For peer review only

BMJ Open

Identifying Naturally Occurring Communities of Primary Care Providers in the English National Health Service in London

Journal:	*BMJ Open*
Manuscript ID	bmjopen-2019-036504.R1
Article Type:	Original research
Date Submitted by the Author:	14-Apr-2020
Complete List of Authors:	Clarke, Jonathan; Imperial College London, Centre for Health Policy; Imperial College London, Centre for Mathematics of Precision Healthcare<br>Beaney, Thomas; Imperial College of Science Technology and Medicine, Department of Primary Care<br>Majeed, Azeem; Imperial College, Primary Care<br>Darzi, Ara; Imperial College London,<br>Barahona, Mauricio; Imperial College London, Centre for Mathematics of Precision Healthcare; Imperial College London, Department of Mathematics
**Primary Subject Heading**:	Health policy
Secondary Subject Heading:	General practice / Family practice, Health informatics, Health services research, Public health
Keywords:	Health policy < HEALTH SERVICES ADMINISTRATION & MANAGEMENT, Organisation of health services < HEALTH SERVICES ADMINISTRATION & MANAGEMENT, PRIMARY CARE, PUBLIC HEALTH

SCHOLARONE™ Manuscripts

I, the Submitting Author has the right to grant and does grant on behalf of all authors of the Work (as defined in the below author licence), an exclusive licence and/or a non-exclusive licence for contributions from authors who are: i) UK Crown employees; ii) where BMJ has agreed a CC-BY licence shall apply, and/or iii) in accordance with the terms applicable for US Federal Government officers or employees acting as part of their official duties; on a worldwide, perpetual, irrevocable, royalty-free basis to BMJ Publishing Group Ltd ("BMJ") its licensees and where the relevant Journal is co-owned by BMJ to the co-owners of the Journal, to publish the Work in this journal and any other BMJ products and to exploit all rights, as set out in our licence.

The Submitting Author accepts and understands that any supply made under these terms is made by BMJ to the Submitting Author unless you are acting as an employee on behalf of your employer or a postgraduate student of an affiliated institution which is paying any applicable article publishing charge ("APC") for Open Access articles. Where the Submitting Author wishes to make the Work available on an Open Access basis (and intends to pay the relevant APC), the terms of reuse of such Open Access shall be governed by a Creative Commons licence – details of these licences and which Creative Commons licence will apply to this Work are set out in our licence referred to above.

Other than as permitted in any relevant BMJ Author's Self Archiving Policies, I confirm this Work has not been accepted for publication elsewhere, is not being considered for publication elsewhere and does not duplicate material already published. I confirm all authors consent to publication of this Work and authorise the granting of this licence.

For peer review only

Title:

Identifying Naturally Occurring Communities of Primary Care Providers in the English National Health Service in London

Authors:

Jonathan Clarke, Thomas Beaney, Azeem Majeed, Ara Darzi, Mauricio Barahona

Jonathan M Clarke
Clinical Research Fellow, Institute of Global Health Innovation, Imperial College London
Level 1, Faculty Building, South Kensington Campus, London, UK, SW7 2NA
ORCID ID: 0000-0003-1495-7746

Thomas E Beaney
Academic Clinical Fellow, Department of Primary Care and Public Health, Imperial College London
Level 2, Faculty Building, South Kensington Campus, London, SW7 2AZ, UK

Azeem Majeed
Professor of Primary Care, Department of Primary Care and Public Health, Imperial College London
Level 2, Faculty Building, South Kensington Campus, London, SW7 2AZ, UK

Ara W Darzi
Professor of Surgery, Institute of Global Health Innovation, Imperial College London
Level 1, Faculty Building, South Kensington Campus, London, SW7 2NA, UK

Mauricio Barahona
Professor of Mathematics, Department of Mathematics, Imperial College London
Huxley Building, South Kensington Campus, London, SW7 2AZ, UK

Correspondence to:
Jonathan Clarke
Clinical Research Fellow, Institute of Global Health Innovation, Imperial College London
Level 1, Faculty Building, South Kensington Campus, London, UK, SW7 2NA
j.clarke@imperial.ac.uk,
+44 (0) 7792 690037

Word Count: 3,416

For peer review only

Abstract

Objectives

Primary Care Networks (PCNs) are a new organisational hierarchy with wide-ranging responsibilities introduced in the National Health Service (NHS) Long Term Plan. The vision is that PCNs should represent 'natural' communities of general practices (GP practices) collaborating at scale and covering a geography that fits well with practices, other healthcare providers and local communities. Our study aims to identify natural communities of GP practices based on patient registration patterns using Markov Multiscale Community Detection, an unsupervised network-based clustering technique to create catchments for these communities.

Design

Retrospective observational study using Hospital Episode Statistics - patient-level administrative records of attendances to hospital.

Setting

General practices in the 32 Clinical Commissioning Groups of Greater London

Participants

All adult patients resident in and registered to a GP practice in Greater London that had one or more outpatient encounters at NHS hospitals between 1st April 2017 and 31st March 2018.

Main outcome measures

The allocation of GP practices in Greater London to PCNs based on the registrations of patients resident in each Lower Layer Super Output Area (LSOA) of Greater London. The population size and coverage of each proposed PCN.

Results

3,428,322 unique patients attended 1,334 GPs in 4,835 LSOAs in Greater London. Our model grouped 1,291 GPs (96.8%) and 4,721 LSOAs (97.6%) into 165 mutually exclusive PCNs. Median PCN list size was 53,490, with a lower quartile of 38,079 patients and an upper quartile of 72,982 patients. A median of 70.1% of patients attended a GP within their allocated PCN, ranging from 44.6% to 91.4%.

Conclusions

With PCNs expected to take a role in population health management and with community providers expected to reconfigure around them, it is vital to recognise how PCNs represent their communities.

For peer review only

Our method may be used by policy-makers to understand the populations and geography shared between networks.

For peer review only

Strengths and limitations of this study

- In the absence of data-driven approaches, Primary Care Networks have formed through interpersonal relationships between practices rather than through an understanding of the distribution of their registered patients.
- This study uses Markov Multiscale Community Detection, a data-driven, unsupervised clustering method, to identify ‘naturally occurring’ communities of GP practices to collectively form 165 Primary Care Networks across London.
- In doing so, this technique produces PCNs which are most representative of the spatial communities of patients for whom PCNs provide care.
- NHS England have proposed that PCNs should contain 30,000 to 50,000 patients restricted to a single Clinical Commissioning Group, however we find this may not represent patterns of care delivery in an urban setting.
- The use of Hospital Episode Statistics ensures that the obtained PCNs are related to secondary care utilisation; yet, on the other hand, these PCNs may not reflect patients who rarely use healthcare services but remain registered to a GP practice.

For peer review only

Introduction

The introduction of Primary Care Networks in the National Health Service (NHS) Long Term Plan in 2019 marks one of the biggest changes to general practice in England.[1] Since the NHS was formed, general practice has seen significant evolution from predominantly single-handed GPs, to large-scale businesses incorporating multi-disciplinary teams. This trend has resulted in a corresponding decline over time in the number of practices to 6,984 practices in England in March 2019 with a corresponding increase in the average list size to 8,550 patients.[2] In parallel, different organisational hierarchies have developed and coexist, linking together practices within a defined geography. Currently, many GP practices collaborate informally as Federations, while all GP practices are now part of larger Clinical Commissioning Groups (CCGs). More recently, Sustainability and Transformation Partnerships have formed through collaboration between CCGs. These structures form part of a move towards integrated systems of care, with the aim of reducing the complexity and fragmentation within healthcare delivery. There is a lack of robust evidence on the effects on patient outcomes, but some evidence indicates that integrated care may improve patient satisfaction and may reduce hospital admission rates and length of stay in older adults.[3,4]

The new NHS Long Term Plan and GP contract announced in 2019 introduced the 'Primary Care Network' (PCN),[1,5,6] a major new organisational structure operating at a population size smaller than the CCG. PCNs are designed to provide economies of scale, without losing the community focus of general practice. In contrast to the informal arrangement of GP Federations, PCNs represents a policy shift towards more formal collaboration between GP practices at a smaller scale than CCGs. This new organisational structure comes with significant financial incentives—an additional £1.8 billion in the form of Directed Enhanced Services has been earmarked to recruit additional staff and expand the role of primary care.[7] Two of the key roles of PCNs will be to integrate community care providers within the network, and to take on an increasing role in the population health management of their communities. These objectives highlight the importance of properly delineating geographically contiguous areas to ensure the efficiency of services provided by PCNs.[5]

While many practices are already members of networks or federations, or informally collaborate with other GPs, the new organisational structure may not match existing arrangements. According to NHS England, PCNs are specified to be networks of neighbouring practices covering a population of 30,000 to 50,000, which will not cross the boundaries of a CCG, although this is not an absolute requirement.[5] The vision from NHS England is that PCNs serve 'natural communities' and that their boundaries make sense to their practices, community-based providers, and to their local communities.[8] In reality, boundaries that make sense to all three groups may be difficult to harmonise, particularly where patients are free to choose a GP outside their geographical area.

For peer review only - http://bmjopen.bmj.com/site/about/guidelines.xhtml

In this article, we set out an approach to defining communities that conform to the criteria required of PCNs, based only on the registration of patients from a given geographical area to a GP. Our approach uses Markov Multiscale Community Detection (MMCD), which uses Louvain optimisation to detect and obtain robust partitions of a network, without imposing *a priori* the number of partitions that should be produced.[9–11] Briefly, MMCD is an unsupervised graph-based clustering method that exploits how a diffusion process spreads on a network to identify communities of nodes that share information consistently over different time scales. In this case, the nodes are GP practices and the communities correspond to practices that have stronger connections with one another than to other nodes in the network. These techniques have previously been applied to the organisation of planned orthopaedic care in England[12] and, in other disciplines, to identify communities within processes as diverse as metabolic networks, transport systems and power grids.[12–16] In this study, we use these unsupervised network analysis techniques to identify underlying communities of GP practices who share patients from the same LSOAs of residence as one another. The ensuing 'GP communities' are thus identified in an unsupervised manner based on patient registration data. In doing so, we seek to produce and understand data-driven PCNs within London that meet the ambitions of policy makers to create 'natural' communities of primary care that represent their local populations.

Methods

All adult patients presenting to outpatient secondary care in England from 1st April 2017 to 31st March 2018 were identified from Hospital Episode Statistics (HES).[17] The Lower Layer Super Output Area (LSOA) of residence of the patient and the unique identifier of their registered GP practice were identified from these records. LSOAs are mutually exclusive, collectively exhaustive geographic fine-scale census divisions defined by the UK Office for National Statistics. There are 4,835 LSOAs in London with a mean population of 1,842 people. LSOAs are therefore similar in scale to Census Block Groups in the Unites States. The LSOA is the most granular geographic division which can be readily mapped to Hospital Episode Statistics.

In cases where a patient was registered at more than one LSOA and GP practice combination within the one-year time period of our study, the record with the highest frequency for that individual was chosen; where these were tied, the most recent combination was chosen. GP practices contributing fewer than 100 patients were excluded.

In order to quantify the extent of overlap between areas covered by different GP practices in London, the equivalent market size (EMS) of each LSOA was calculated as the reciprocal of the Herfindahl Hirschman Index (Equations 1 and 2):

For peer review only

$$HHI_i = \sum_{j=1}^{N} s_{ij}^2 \qquad (1)$$

Equation 1: Herfindahl-Hirschman Index of LSOA i. Here s_{ij} is the proportion of patients from LSOA i registered to GP practice j, and N is the number of GP practices in the dataset.

$$EMS_i = 1/HHI_i \qquad (2)$$

Equation 2: Equivalent Market Size of LSOA i is calculated as the reciprocal of the Herfindahl-Hirschman Index of LSOA i.

The EMS represents the number of GP practices to which members of an LSOA would be registered if each practice occupied an equal market share of registrations. It therefore provides an estimate of the concentration of the market for primary care registrations within each LSOA.[18–20] The probability of a patient from each LSOA being registered to a GP practice was calculated, and the cosine similarity matrix between all pairs of GP practices was computed (Equation 3).

$$similarity_{AB} = \frac{\sum_{i=1}^{n} \boldsymbol{A}_i \boldsymbol{B}_i}{\sqrt{\sum_{i=1}^{n} \boldsymbol{A}_i^2}\sqrt{\sum_{i=1}^{n} \boldsymbol{B}_i^2}} \qquad (3)$$

Equation 3: Calculation of the cosine similarity between two GP practices, A and B. Here A_i is the proportion of patients registered to GP practice A resident in LSOA i; B_i is the proportion of patients registered to GP practice B resident in LSOA i; and n is the total number of LSOAs in the dataset.

Equation 3 defines one of the entries of a similarity matrix between all GP practices. This similarity matrix can be thought of as the adjacency matrix of a network connecting GPs to one another with weights reflecting the similarity of their patterns of patient registration across LSOAs, i.e., GP practices with similar LSOA patient profiles are strongly connected. This dense network was sparsened using the Relaxed Minimum Spanning Tree (RMST) technique, a method used elsewhere in applied network science to sparsen a dense, inhomogeneous network to preserve both local and global connectivity within a network.[21–23] The sparsened network was subsequently partitioned using Markov Multiscale Community Detection (MMCD) to produce partitions of the GPs according to shared patterns of registration from LSOAs. Each of these partitions corresponds to a 'natural' community directly derived from the registration data.[9,10,12,24]

MMCD was performed over a range of 400 Markov times from 0.01 to 1 and over a range of 40 RMST pruning parameters from 2.1 to 6.0. Each of these 16,000 models was optimised 500 times and the most

For peer review only

common network partition for each model was selected. Scanning across the range of pruning parameters revealed relative maxima with respect to our coverage function (described below), with clear surrounding margins of suboptimal values.

The geographic co-ordinates of each GP practice were identified from their registered postcode. For each of the 16,000 partitions produced, the pairwise geographic straight-line distances between all practices within each PCN was calculated. Where the median distance from a practice to all other practices within a PCN community was more than four times the median pairwise distance between all practices within the PCN community, this practice was excluded as a spatial outlier. The number of practices within each PCN community was calculated and the proportion of practices contained within the polygon drawn around the outer geographic limits of the practices comprising the PCN community was calculated.

PCN communities where the number of practices was less than three or greater than twenty, or where the percentage of practices spatially located within the outer spatial limits (defined by the polygon drawn around each practice coordinate) of the PCN community was more than 25% were excluded. The total number of practices present in the remaining PCN communities was calculated. The partition with the highest number of included GP practices was taken as the optimal partition.

For this optimal partition, the GP practice name, location and CCG were linked by practice code, using data from NHS Digital. Practice list sizes as of March 2018 were also linked using data from NHS Digital.[2] Where a practice list size was not available, such as in cases where a practice had closed or merged over the time period, list sizes as of April 2017 were used.[25]

LSOAs were subsequently assigned to a PCN community based on the PCN to which the highest number of patients within a given LSOA were registered. GP practices were mapped along with their corresponding assigned community allocation and LSOA boundaries. The proportion of patients resident within the same PCN as their registered GP was calculated for each PCN.

Patient and Public Involvement (PPI) Statement

The data on which this study is based was granted following review by a panel including patient and lay representatives. Patients were not invited to contribute to the writing or editing of this document for readability or accuracy.

Results

A total of 3,428,322 unique adult patients resident in London attended a total of 20,173,937 outpatient appointments to NHS hospitals between 1st April 2017 and 31st March 2018 (Figure 1). All 4,835

For peer review only

LSOAs in London were represented. A total of 1,334 GP practices were identified within London belonging to a total of 32 Clinical Commissioning Groups.

Residents in the same LSOA in London were generally registered to a wide range of GP practices. Figure 2 demonstrates the equivalent market size of LSOAs across London with respect to GP practices. A wide range of equivalent market sizes (EMS_i) was seen, from 1.1 to 20.6. 185 LSOAs (3.8%) had an equivalent market size of less than two GP practices, consistent with primary care provision by a single dominant GP practice. The median equivalent market size across London was 4.9 GP practices per LSOA, while 259 LSOAs (5.4%) had an equivalent market size of more than ten GP practices. The median LSOA-level equivalent market sizes of CCGs in London ranged from 3.2 to 7.0. Overall, the median equivalent market size for LSOAs north of the river Thames was 23% higher than those south of the river (5.3 vs. 4.3).

An optimal configuration of GP communities was found at an RMST parameter of 5.5 and Markov time of 0.054. For this optimal clustering, only 43 GP practices (out of a total of 1,334) were unassigned to a community according to our criteria: 28 practices lay in 20 communities with only 1-2 practices, and a further 15 practices were spatial outliers. Collectively, these 43 practices were the modal provider of primary care for 114 LSOAs with a total population of 187,101 (2.3%). Our optimal partition consisted of 165 PCNs grouping 1,291 practices, which cover 4,721 LSOAs in London and 97.7% of the estimated London population. A map of this optimal configuration, displaying GP practices superimposed on the LSOAs assigned to each PCN is shown in Figure 3. The excluded communities, represented by dashed lines, are predominantly situated peripherally or bordering the river Thames.

The PCNs ranged in size from 3 to 18 practices with a median of 8. Median list size of PCNs was 53,490 patients (ranging from 14,574 to 176,982 patients, with a lower quartile of 38,079 and upper quartile of 72,982 patients). Around two thirds (67.9%) of PCNs contained practices from only one CCG and the remaining PCNs contained practices from either two (23.0%) or three (9.1%) CCGs.

Across all 4,721 LSOAs, the median percentage of patients registered to GP practices located within their allocated PCN was 73.7%, ranging from a minimum of 24.8% to a maximum 98.6% (Figure 4, left). Across the 165 PCNs, a median of 70.1% of patients within the PCN catchment area were registered to a GP practice within the PCN, ranging from a minimum of 44.6% to a maximum of 91.4% (Figure 4, right), and this did not differ between PCNs that did or did not cross CCG boundaries (70.0% vs. 70.2%, two-tailed Mann-Whitney U-test, $p = 0.36$). The median Herfindahl-Hirschman Index of the LSOAs was 0.57, and there was a median equivalent market size of 1.75 PCNs for each LSOA.

Discussion

For peer review only

As the health system develops to prioritise integrated and collaborative working, we need to improve our understanding of the relationships between providers and patients. With the requirement for GPs to form larger organisational units in the form of PCNs, there is a need to quantify how representative these communities are of their populations. The techniques used in our study provide an unsupervised, data-driven means of producing mutually exclusive PCNs formed by bringing together GP practices that frequently provide care to patients from the same geographic regions. In doing so, it makes no assumptions regarding geography or organisational hierarchies and produces 'natural' communities. Using this technique, we showed that despite having no prior geographic knowledge, 97.7% of the population of London may be assigned to a PCN of appropriate size and which are spatially consistent.

The composition of PCNs

The factors that determine practices joining a PCN are complex and rely on the interpersonal and professional relationships between GPs as much as the shared relationships in registration of the local population. Furthermore, pre-defined boundaries cannot be ignored, with local authorities and CCGs taking responsibility for commissioning for a given geographical area and registered patients. However, networks must work for their population, and existing tools to guide decision-making are limited. While most practices have now joined PCNs, it is likely that boundaries will change as practices move in and out of PCNs, reflecting the complex dynamics of a system which needs to work for the population and providers.

In the context of the PCNs that are forming across London, our findings may in the future be directly compared with the actual PCNs that form. While it is hoped that the PCNs suggested by this study conform to those being formed across London, identifying discrepancies between the two conformations may offer a means of identifying PCNs which may not adequately represent local patterns of patient registration, and therefore may benefit from reorganisation. Secondly, where practices struggle to align with one another to form PCNs, the findings of this study offer a tool by which to propose suitable PCN structures.

Our findings also raise questions regarding the optimal size and configuration of PCNs across London. Our modelling suggests that the 30,000 to 50,000 recommended list size for PCNs may be too restrictive. The finding of a median list size for our optimal configuration of 53,490 in London, with an interquartile range from 38,000 to 73,000 patients suggests that the current recommended range may not permit larger networks to form where underlying patient registration patterns would favour this. A wider range of list sizes also suggests that greater variability in PCN size should be allowed depending on local need. Similarly, while a clear majority of the PCNs we found (67.9%) are formed from practices within a single CCG, almost one third contain practices in two or three CCGs, suggesting that there should be

For peer review only

flexibility in conformity to CCG boundaries. However, there may be additional problems for a PCN containing practices from more than one CCG where priorities do not align.

The role of PCNs

If PCNs take on a role in population health management, and community services are required to reconfigure to match the footprints of PCNs,[26,27] then the need to understand the demographics and geography of PCNs becomes critical. Through our network analysis method, we defined an approach to assign catchment areas and estimate the catchment population for each PCN. We found that on average, almost one third of the population living within the PCN catchment area attended a practice outside of the PCN, which was as high as half of the population for some PCNs. Understanding the dynamics of registration patterns in the local population could thus have significant implications on the design of place-based services.

Population dispersion is likely to be a greater problem in a densely populated urban area such as London, where there are extensive transport links and a greater choice of providers within a shorter commuting distance. However, our estimates, which by design are modelled to represent optimal patterns of registration, are likely to overestimate coverage of the PCN compared to those PCNs that have already formed. The opportunity for patients to retain registration with a practice after moving outside of its historic catchment postcodes may reduce coverage of a PCN where a fixed relationship to a discrete geographic area is required.

The emergence of digital NHS general practice, in the form of GP at Hand and its peers which allow patients access to video consultations as the first point of contact, disrupts the notion of a ‘place-based’ relationship between primary care providers and geographic communities of patients. Currently, the registered population for such services is disproportionately of working age, with 98.5% of registrants between 20-64 years of age, compared to a London-wide estimate of 75.3%.[28,29] The proportion of patients with one or more long term conditions using this service is almost half that of London-wide estimates (10.6% compared to 18.3%).[28,30] Therefore, patients registered with digital general practices are less likely to require the place-based services and community integration which underpins the establishment of Primary Care Networks.

As such innovations in registration and GP provision mature and potentially scale across the health system, the significant differences between patients using digital primary care services and traditional general practices may undermine efforts to enforce relationships between primary care providers and discrete geographic communities of patients. This trend may signal the emergence of a differentiated system of primary care, where those with low care needs are served by essentially rootless digital primary care providers and those with higher care needs are attended to by well-integrated, accessible

For peer review only

primary care providers with a nearby physical presence. In such cases, the creation of Primary Care Networks by providers of digital primary care may be orthogonal to the underlying ethos of vertical integration and investment in relationships with community care providers that underlies the current policy.

Limitations

One of the main limitations of our analysis is in use of secondary care, rather than primary care data. While HES represented 3.4 million patients in London, this covers less than half of the estimated population of 8.8 million.[27,31] Patients in HES have, by definition, used secondary care services at least once over the study period, and may not be representative of the whole population registered with primary care. Older people, and those with a higher number of chronic conditions are more likely to be represented in HES, and these patients may have different criteria in choosing a GP. Use of complete lists of GP practice registrations obtained from practices and covering the whole of London, would be preferable, but is not readily available and was outside the scope of the current study.

A limitation with our method was that the optimal configuration was unable to fit every practice in London to a PCN, with 43 (out of 1,334) unassigned as a result of our selection criteria. 15 practices were unassigned as spatial outliers, which is to say that their median distance to all other practices in their network was more than four times greater than the median pairwise distance between all practices in the network. These rare instances may reflect the statistical noise of the modelling technique which is agnostic to the spatial proximity of providers to one another. A further 28 practices were unassigned due to their proposed PCNs containing fewer than three practices. In these cases, allocation of unassigned practices in collaboration with practices and commissioners, to nearby larger PCNs could be an appropriate solution to ensure complete allocation of practices to PCNs. The finding that many unassigned practices were near the periphery of London suggests a boundary effect where the exclusion of practices and the population outside of London may have affected the model in these regions.

Conclusion

As health systems adapt towards closer integration across services, network analysis offers a data-driven and unbiased means of understanding the connections between Primary Care Networks and their patients. Our findings demonstrate that GP practices may be combined into communities reflecting their underlying populations in accordance with the specification of PCNs. At a time when integration of community, primary and secondary care is being prioritized, concurrently, place-based primary care anchored in the local community is increasingly being challenged with the growth of online GP consultation providers, such as that provided by GP at Hand in London. Upscaling primary care into larger networks has the potential to weaken further the ties between providers and their communities. There is a pressing need to better understand how these networks will represent their geographies and

For peer review only

patients, to identify who may gain and who may lose out, and ensure a well-intentioned policy does not widen inequalities in health.

For peer review only

Author Contributions

JC and TB were involved in all aspects of the study. MB was involved in the development of the methodology and assisted in the formal analysis. MB, AM and AD were involved in the conceptualization of the study and in the reviewing and editing of the draft. JC has had access to all the data in the study and all authors had final responsibility for the decision to submit for publication. JC attests that all listed authors meet authorship criteria and that no others meeting the criteria have been omitted.

Acknowledgements and Competing Interests

All authors have completed the ICMJE uniform disclosure form at www.icmje.org/coi_disclosure.pdf and declare: JC, TB, AD and MB have no competing interests to declare. AM is a GP Principal in an NHS general practice.

Funding

This article is independent research supported by grants from The Peter Sowerby Foundation and the National Institute for Health Research (NIHR) Imperial Patient Safety and Translational Research Centre (PSTRC) PSTRC_2016_004. Infrastructure support for this work was provided by the NIHR Imperial Biomedical Research Centre (BRC) 1215-20013. TB is supported by a National Institute for Health Research (NIHR) Academic Clinical Fellowship. TB acknowledges support from the NIHR Imperial Biomedical Research Centre (BRC) and the NIHR Applied Research Collaboration for NW London. AM is supported by the NIHR Applied Research Collaboration for NW London. MB acknowledges support from EPSRC grant EP/N014529/1 supporting the EPSRC Centre for Mathematics of Precision Healthcare. Data management was provided by the Big Data and Analytical Unit (BDAU) at the Institute of Global Health Innovation (IGHI), Imperial College London. The views expressed in this publication are those of the author(s) and not necessarily those of the NHS, the National Institute for Health Research or the Department of Health.

For peer review only

Data Sharing Statement

Data used in this study were obtained from NHS Digital for the purpose of this work and may only be accessed through direct application to NHS Digital. Patient-level data is required for the analyses conducted, and therefore sharing of data pertaining to this study is not possible. Data for primary care network assignments are available from the authors on request.

Dissemination declaration

Data as to GP practice-level allocation to putative Primary Care Networks will be made publicly available on www.healthdatascience.co.uk and have been disseminated to local primary care providers and commissioners.

Transparency declaration

The lead author affirms that this manuscript is an honest, accurate, and transparent account of the study being reported; that no important aspects of the study have been omitted; and that any discrepancies from the study as planned have been explained.

Statement of Ethical Approval

This study received local ethical approval through the Imperial College Research Ethics Committee (17IC4178).

For peer review only

Figures:

Figure 1: Algorithm for participant selection from Hospital Episode Statistics

Figure 2: Choropleth map representing the Equivalent Market Size of general practices for all LSOAs in London, as calculated in Equation 2. The histogram shows the frequency distribution of Equivalent Market Sizes for the 4,835 LSOAs in London. Higher values indicate more dilute markets with patients registered to a wider range of GP practices.

Figure 3: Optimal configuration of general practice communities in London obtained from our model, with LSOAs assigned to most common practice of registration. Dashed lines represent unassigned communities. Circles represent the location of GP practices, and are coloured according to the community they are assigned. White circles indicate the 43 GP practices that are not assigned to any community.

Figure 4: Left: Choropleth map representing the proportion of patients within each LSOA attending the modal PCN. Right: Choropleth map representing the proportion of patients within the LSOAs assigned to each PCN attending a practice within the PCN. Dashed lines represented unassigned communities.

For peer review only

References:

1. NHS England. *The NHS Long Term Plan*. (2019).
2. NHS Digital. Patients Registered at a GP Practice, March 2018. *Online, NHS Digital* (2018).
3. Baxter, S. *et al. Understanding new models of integrated care in developed countries: a systematic review*. (2018). doi:10.3310/hsdr06290
4. Liljas, A. E. M., Brattström, F., Burström, B., Schön, P. & Agerholm, J. Impact of Integrated Care on Patient-Related Outcomes Among Older People - A Systematic Review. *Int. J. Integr. Care* **19**, 6 (2019).
5. NHS England & British Medical Association. *Investment and evolution : A five-year framework for GP contract reform to implement The NHS Long Term Plan*. (2019).
6. Baird, B. Primary care networks explained. *The King's Fund* (2019).
7. NHS England. *Network Contract Directed Enhanced Service*. (2019).
8. NHS England. Primary care networks. (2019).
9. Delvenne, J.-C., Yaliraki, S. N. & Barahona, M. Stability of graph communities across time scales. *Proc. Natl. Acad. Sci. U. S. A.* **107**, 12755–60 (2010).
10. Delvenne, J.-C., Schaub, M. T., Yaliraki, S. N. & Barahona, M. The Stability of a Graph Partition: A Dynamics-Based Framework for Community Detection. in *Dynamics On and Of Complex Networks, Volume 2*. 221–242 (Birkhäuser, New York, NY, 2013). doi:10.1007/978-1-4614-6729-8_11
11. Lambiotte, R., Delvenne, J.-C. & Barahona, M. Random Walks, Markov Processes and the Multiscale Modular Organization of Complex Networks. *IEEE Trans. Netw. Sci. Eng.* **1**, 76–90 (2014).
12. Schaub, M. T., Delvenne, J. C., Yaliraki, S. N. & Barahona, M. Markov dynamics as a zooming lens for multiscale community detection: Non clique-like communities and the field-of-view limit. *PLoS One* **7**, 1–20 (2012).
13. Schaub, M. T., Lehmann, J., Yaliraki, S. N. & Barahona, M. Structure of complex networks: Quantifying edge-to-edge relations by failure-induced flow redistribution. *Netw. Sci.* **2**, 1–24 (2014).
14. Beguerisse-Díaz, M., Bosque, G., Oyarzún, D., Picó, J. & Barahona, M. Flux-dependent graphs for metabolic networks. 1–25 (2016).
15. Clarke, J. M., Barahona, M. & Darzi, A. W. Defining Hospital Catchment Areas Using Multiscale Community Detection: A Case Study for Planned Orthopaedic Care in England. *bioRxiv* 619692 (2019). doi:10.1101/619692
16. Schaub, M. T., Lambiotte, R. & Barahona, M. Encoding dynamics for multiscale community detection: Markov time sweeping for the map equation. *Phys. Rev. E - Stat. Nonlinear, Soft Matter Phys.* **86**, 1–10 (2012).

For peer review only

17. NHS Digital. Hospital Episode Statistics (HES). (2018). Available at: https://digital.nhs.uk/data-and-information/data-tools-and-services/data-services/hospital-episode-statistics.

18. Clarke, J. M., Warren, L. R., Arora, S., Barahona, M. & Darzi, A. W. Guiding interoperable electronic health records through patient-sharing networks. *npj Digit. Med.* **1**, 65 (2018).

19. Rhoades, S. A. Market share inequality, the HHI, and other measures of the firm-composition of a market. *Rev. Ind. Organ.* **10**, 657–674 (1995).

20. Feng, Y. *et al.* Association between market concentration of hospitals and patient health gain following hip replacement surgery. *J. Health Serv. Res. Policy* **20**, 11–7 (2015).

21. Amor, B. R. C. *et al.* Community detection and role identification in directed networks: Understanding the twitter network of the care.data debate. in *Dynamic Networks and Cyber-Security* **1**, 111–136 (World Scientific Publishing Co. Pte. Ltd., 2016).

22. Altuncu, M. T., Mayer, E., Yaliraki, S. N. & Barahona, M. From free text to clusters of content in health records: an unsupervised graph partitioning approach. *Appl. Netw. Sci.* **4**, 2 (2019).

23. Beguerisse-Diaz, M., Vangelov, B. & Barahona, M. Finding role communities in directed networks using Role-Based Similarity, Markov Stability and the Relaxed Minimum Spanning Tree. *2013 IEEE Glob. Conf. Signal Inf. Process. Glob. 2013 - Proc.* 937–940 (2013). doi:10.1109/GlobalSIP.2013.6737046

24. Lambiotte, R., Delvenne, J.-C. & Barahona, M. Laplacian Dynamics and Multiscale Modular Structure in Networks. 1–29 (2008). doi:10.1109/TNSE.2015.2391998

25. NHS Digital. Numbers of Patients Registered at a GP Practice - April 2017. *Online, NHS Digital* (2017).

26. Murray, R. Primary care networks and community health services: further, faster! *The King's Fund* (2019).

27. Murray, R. Primary care networks and the NHS long-term plan: the new player on the pitch. *The King's Fund* (2019).

28. Care Quality Commission. *GP at hand: Inspection report*.

29. Office for National Statistics. Mid-year Population Estimates 2019 - Office for National Statistics. Available at: https://www.ons.gov.uk/peoplepopulationandcommunity/populationandmigration/populationestimates. (Accessed: 26th October 2019)

30. NHS England. *London - A Call to Action*. (2013).

31. Greater London Authority. GLA population and Household Projections - Central Trend (population, 2017-based). (2017).

For peer review only

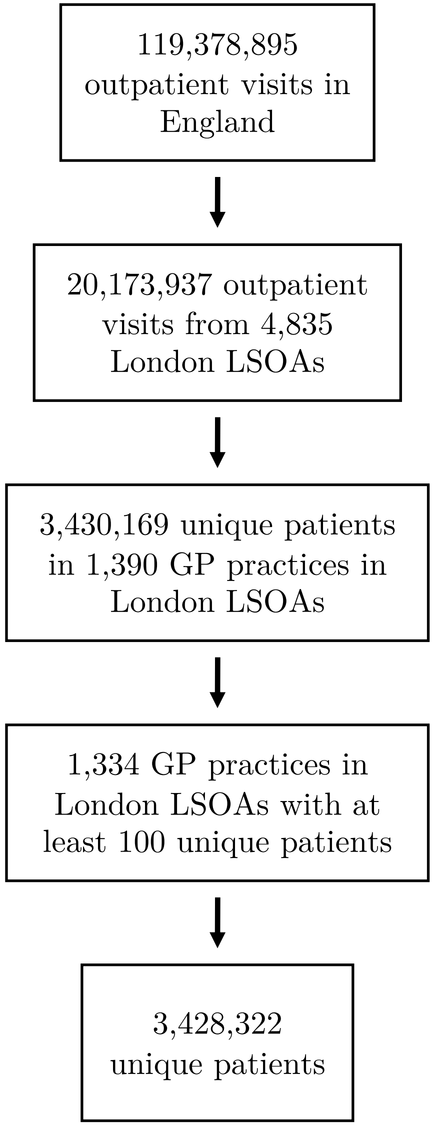
119,378,895 outpatient visits in England
20,173,937 outpatient visits from 4,835 London LSOAs
3,430,169 unique patients in 1,390 GP practices in London LSOAs
1,334 GP practices in London LSOAs with at least 100 unique patients
3,428,322 unique patients

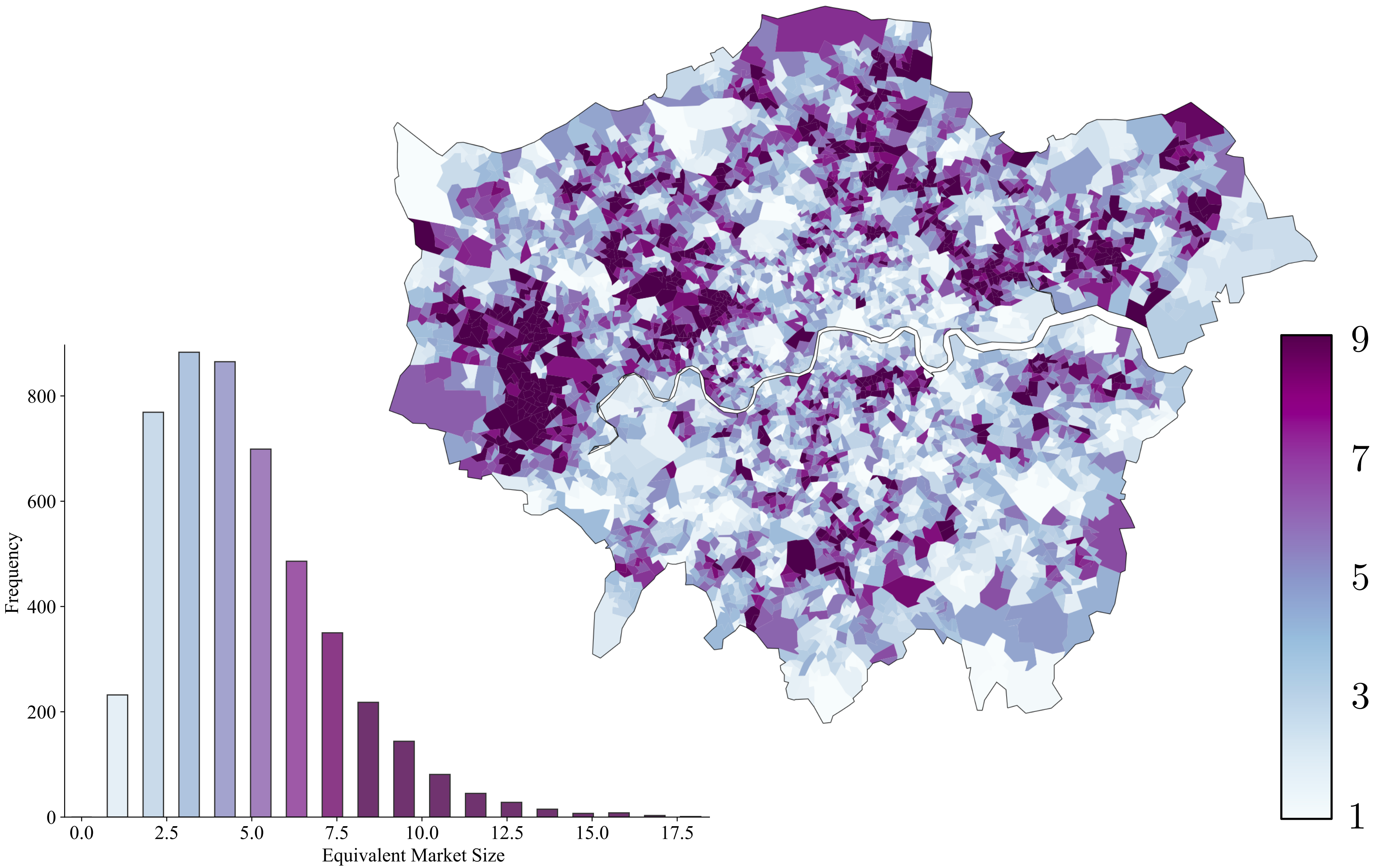
800
600
400
200
0
Frequency
0.0
2.5
5.0
7.5
10.0
12.5
15.0
17.5
Equivalent Market Size
9
7
5
3
1

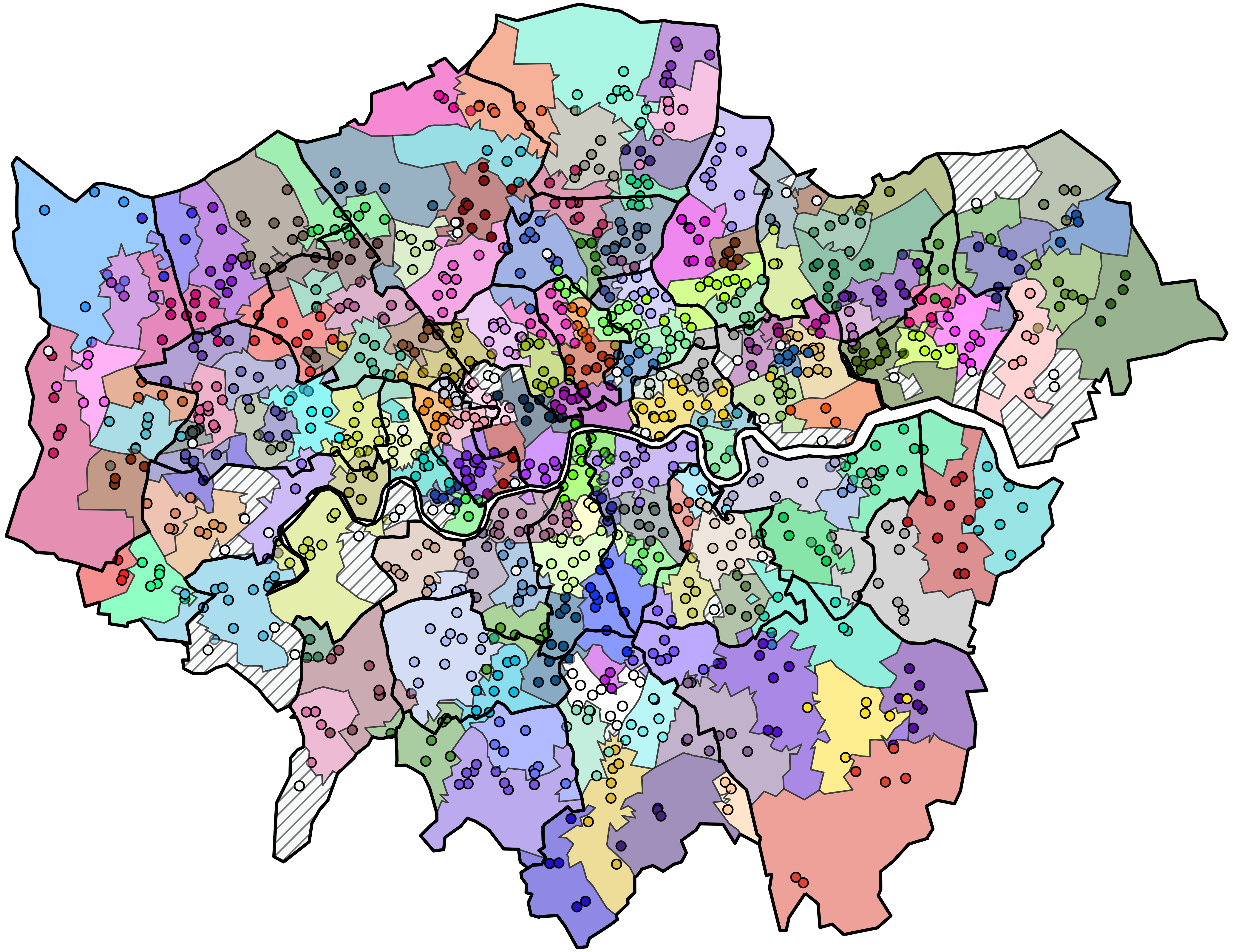

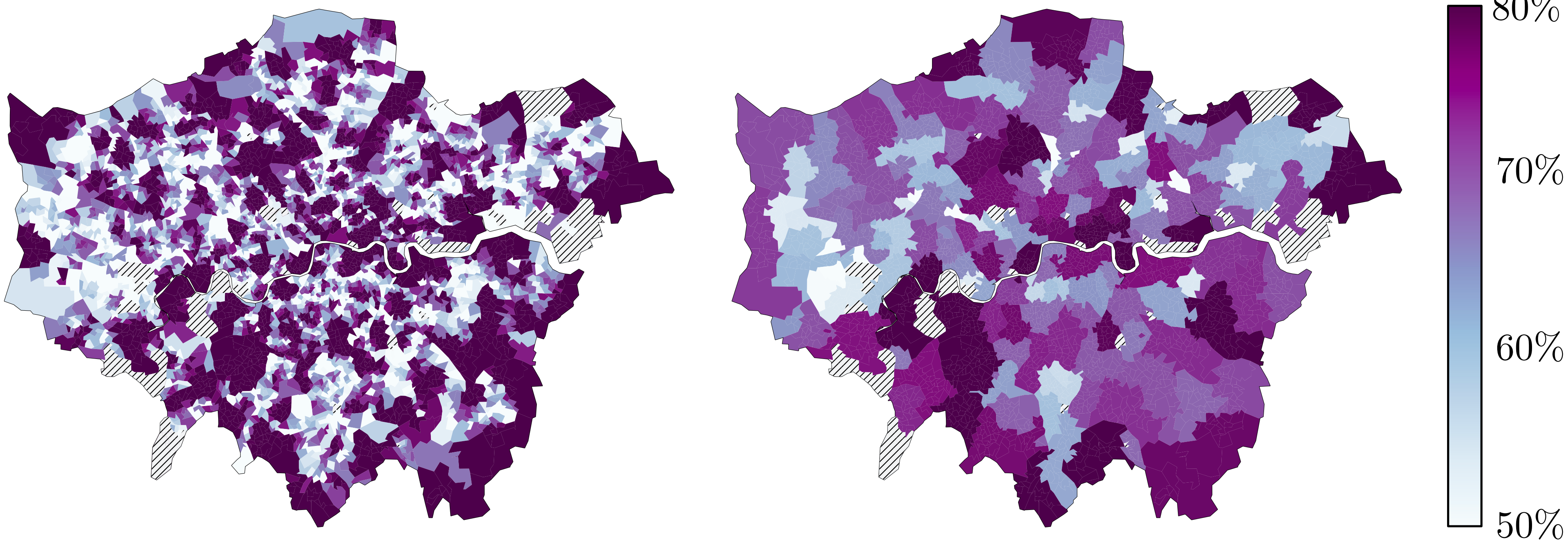
80%
70%
60%
50%

The RECORD statement – checklist of items, extended from the STROBE statement, that should be reported in observational studies using routinely collected health data.

	Item No.	STROBE items	Location in manuscript where items are reported	RECORD items	Location in manuscript where items are reported
**Title and abstract**					
	1	(a) Indicate the study's design with a commonly used term in the title or the abstract (b) Provide in the abstract an informative and balanced summary of what was done and what was found	Abstract	RECORD 1.1: The type of data used should be specified in the title or abstract. When possible, the name of the databases used should be included.<br><br>RECORD 1.2: If applicable, the geographic region and timeframe within which the study took place should be reported in the title or abstract.<br><br>RECORD 1.3: If linkage between databases was conducted for the study, this should be clearly stated in the title or abstract.	Abstract (methods)<br><br>Abstract (setting / participants)<br><br>N/A
**Introduction**					
Background rationale	2	Explain the scientific background and rationale for the investigation being reported	Introduction		
Objectives	3	State specific objectives, including any prespecified hypotheses	Introduction		
**Methods**					
Study Design	4	Present key elements of study design early in the paper	Methods		
Setting	5	Describe the setting, locations, and relevant dates, including periods of recruitment, exposure, follow-up, and data collection	Methods		

For peer review only

Participants	6	*(a) Cohort study* - Give the eligibility criteria, and the sources and methods of selection of participants. Describe methods of follow-up<br>*Case-control study* - Give the eligibility criteria, and the sources and methods of case ascertainment and control selection. Give the rationale for the choice of cases and controls<br>*Cross-sectional study* - Give the eligibility criteria, and the sources and methods of selection of participants<br><br>*(b) Cohort study* - For matched studies, give matching criteria and number of exposed and unexposed<br>*Case-control study* - For matched studies, give matching criteria and the number of controls per case	N/A<br>N/A<br>Methods<br>N/A<br>N/A	RECORD 6.1: The methods of study population selection (such as codes or algorithms used to identify subjects) should be listed in detail. If this is not possible, an explanation should be provided.<br><br>RECORD 6.2: Any validation studies of the codes or algorithms used to select the population should be referenced. If validation was conducted for this study and not published elsewhere, detailed methods and results should be provided.<br><br>RECORD 6.3: If the study involved linkage of databases, consider use of a flow diagram or other graphical display to demonstrate the data linkage process, including the number of individuals with linked data at each stage.	Methods<br><br>Validation of Markov Multiscale Community Detection cited<br><br>N/A
Variables	7	Clearly define all outcomes, exposures, predictors, potential confounders, and effect modifiers. Give diagnostic criteria, if applicable.	Methods	RECORD 7.1: A complete list of codes and algorithms used to classify exposures, outcomes, confounders, and effect modifiers should be provided. If these cannot be reported, an explanation should be provided.	Methods
Data sources/ measurement	8	For each variable of interest, give sources of data and details of methods of assessment (measurement).<br>Describe comparability of assessment methods if there is more than one group	Methods		

For peer review only

Bias	9	Describe any efforts to address potential sources of bias	Discussion		
Study size	10	Explain how the study size was arrived at	Methods		
Quantitative variables	11	Explain how quantitative variables were handled in the analyses. If applicable, describe which groupings were chosen, and why	Methods		
Statistical methods	12	(a) Describe all statistical methods, including those used to control for confounding<br>(b) Describe any methods used to examine subgroups and interactions<br>(c) Explain how missing data were addressed<br>(d) *Cohort study* - If applicable, explain how loss to follow-up was addressed<br>*Case-control study* - If applicable, explain how matching of cases and controls was addressed<br>*Cross-sectional study* - If applicable, describe analytical methods taking account of sampling strategy<br>(e) Describe any sensitivity analyses	N/A<br>Methods<br>Discussion<br>N/A<br>N/A<br>Methods, Discussion<br>N/A		
Data access and cleaning methods		..		RECORD 12.1: Authors should describe the extent to which the investigators had access to the database population used to create the study population.	Declaration of interests

				RECORD 12.2: Authors should provide information on the data cleaning methods used in the study.	Methods
Linkage		..		RECORD 12.3: State whether the study included person-level, institutional-level, or other data linkage across two or more databases. The methods of linkage and methods of linkage quality evaluation should be provided.	Methods
**Results**					
Participants	13	(a) Report the numbers of individuals at each stage of the study (*e.g.*, numbers potentially eligible, examined for eligibility, confirmed eligible, included in the study, completing follow-up, and analysed)<br>(b) Give reasons for non-participation at each stage.<br>(c) Consider use of a flow diagram	Results<br>Discussion<br>Figure 1	RECORD 13.1: Describe in detail the selection of the persons included in the study (*i.e.,* study population selection) including filtering based on data quality, data availability and linkage. The selection of included persons can be described in the text and/or by means of the study flow diagram.	Methods, Figure 1
Descriptive data	14	(a) Give characteristics of study participants (*e.g.*, demographic, clinical, social) and information on exposures and potential confounders<br>(b) Indicate the number of participants with missing data for each variable of interest<br>(c) *Cohort study* - summarise follow-up time (*e.g.*, average and total amount)	Results<br>Results<br>N/A		
Outcome data	15	*Cohort study* - Report numbers of outcome events or summary measures over time<br>*Case-control study* - Report numbers in each exposure	N/A<br>N/A		

For peer review only

		category, or summary measures of exposure<br>*Cross-sectional study* - Report numbers of outcome events or summary measures	Results		
Main results	16	(a) Give unadjusted estimates and, if applicable, confounder-adjusted estimates and their precision (e.g., 95% confidence interval). Make clear which confounders were adjusted for and why they were included<br>(b) Report category boundaries when continuous variables were categorized<br>(c) If relevant, consider translating estimates of relative risk into absolute risk for a meaningful time period	Results<br><br>N/A<br><br>N/A		
Other analyses	17	Report other analyses done—e.g., analyses of subgroups and interactions, and sensitivity analyses	Results		
**Discussion**					
Key results	18	Summarise key results with reference to study objectives	Discussion		
Limitations	19	Discuss limitations of the study, taking into account sources of potential bias or imprecision. Discuss both direction and magnitude of any potential bias	Discussion	RECORD 19.1: Discuss the implications of using data that were not created or collected to answer the specific research question(s). Include discussion of misclassification bias, unmeasured confounding, missing data, and changing eligibility over time, as they pertain to the study being reported.	Discussion
Interpretation	20	Give a cautious overall interpretation of results considering objectives,	Discussion		

For peer review only

		limitations, multiplicity of analyses, results from similar studies, and other relevant evidence			
Generalisability	21	Discuss the generalisability (external validity) of the study results	Discussion		
**Other Information**					
Funding	22	Give the source of funding and the role of the funders for the present study and, if applicable, for the original study on which the present article is based	Included after conclusions of manuscript		
Accessibility of protocol, raw data, and programming code		..		RECORD 22.1: Authors should provide information on how to access any supplemental information such as the study protocol, raw data, or programming code.	Included after conclusions of manuscript

*Reference: Benchimol EI, Smeeth L, Guttmann A, Harron K, Moher D, Petersen I, Sørensen HT, von Elm E, Langan SM, the RECORD Working Committee. The REporting of studies Conducted using Observational Routinely-collected health Data (RECORD) Statement. *PLoS Medicine* 2015; in press.

*Checklist is protected under Creative Commons Attribution (CC BY) license.

For peer review only
